# Personal Health Records: A Systematic Literature Review

**DOI:** 10.2196/jmir.5876

**Published:** 2017-01-06

**Authors:** Alex Roehrs, Cristiano André da Costa, Rodrigo da Rosa Righi, Kleinner Silva Farias de Oliveira

**Affiliations:** ^1^ Programa de Pós-Graduação em Computação Aplicada Universidade do Vale do Rio dos Sinos São Leopoldo Brazil

**Keywords:** personal health records, patient access to records, mobile health, electronic health records, taxonomy

## Abstract

**Background:**

Information and communication technology (ICT) has transformed the health care field worldwide. One of the main drivers of this change is the electronic health record (EHR). However, there are still open issues and challenges because the EHR usually reflects the partial view of a health care provider without the ability for patients to control or interact with their data. Furthermore, with the growth of mobile and ubiquitous computing, the number of records regarding personal health is increasing exponentially. This movement has been characterized as the Internet of Things (IoT), including the widespread development of wearable computing technology and assorted types of health-related sensors. This leads to the need for an integrated method of storing health-related data, defined as the personal health record (PHR), which could be used by health care providers and patients. This approach could combine EHRs with data gathered from sensors or other wearable computing devices. This unified view of patients’ health could be shared with providers, who may not only use previous health-related records but also expand them with data resulting from their interactions. Another PHR advantage is that patients can interact with their health data, making decisions that may positively affect their health.

**Objective:**

This work aimed to explore the recent literature related to PHRs by defining the taxonomy and identifying challenges and open questions. In addition, this study specifically sought to identify data types, standards, profiles, goals, methods, functions, and architecture with regard to PHRs.

**Methods:**

The method to achieve these objectives consists of using the systematic literature review approach, which is guided by research questions using the population, intervention, comparison, outcome, and context (PICOC) criteria.

**Results:**

As a result, we reviewed more than 5000 scientific studies published in the last 10 years, selected the most significant approaches, and thoroughly surveyed the health care field related to PHRs. We developed an updated taxonomy and identified challenges, open questions, and current data types, related standards, main profiles, input strategies, goals, functions, and architectures of the PHR.

**Conclusions:**

All of these results contribute to the achievement of a significant degree of coverage regarding the technology related to PHRs.

## Introduction

### Overview

The physician-patient relationship traditionally consists of the total dependence of the patient on the physician. Physicians need to keep accurate record systems to store information about patients and use the records to make diagnoses and recommendations [1]. In this sense, one important milestone is the use of the electronic health record (EHR). Health records are collections of patient health data, and the EHR is defined as a digital repository of the health status of patients [2-4]. The EHR evolved from a number of electronic methods of storing patients’ health data that became a structured and interoperable approach [5,6]. However, EHRs have some limitations because their records are based entirely on data reported by health care providers [3]. One trend is allowing patients to have access to their own health data, making them the owner of such data [7,8]. Therefore, personal health records (PHRs) emerged from the EHR and are defined as health records related to patient care that are controlled by the patient [6,9]. The PHR can also be defined as a representation of the health information, wellness, and development of a person [10]. The main advantages of the PHR refer to the ability of patients to maintain data on their health. However, many challenges need to be overcome to promote widespread PHR adoption, including how to achieve interoperability using the EHR, implementation costs, privacy, security, and the assessment of the effective benefits that the patient may have [1].

PHRs allow patients to maintain information on their medical conditions, drugs, and behaviors related to self-care and self-monitoring of their health [11]. Nevertheless, access controlled by the patients represents an ever-present concern because it requires a free but safe balance between system customizations, privacy, and security controls [12]. In particular, without the application of security practices, no privacy is available for the data [13-15]. Another possibility is that the PHRs accept data obtained from health-related equipment, such as accelerometers, gyroscopes, wireless scales, wristbands, and smartwatches. The proliferation of these technologies is called the Internet of Things (IoT) [16,17]. Among IoT application domains, health care is one of the most attractive, giving rise to many health-related devices [18]. Data collected from these objects can complement the PHRs and help detect risks to the patients’ health [16]. Nonetheless, existing PHRs have limited intelligence and can only inform a small subset of users’ health care needs [19]. In addition, processing PHR data automatically and combining data from sensors with stored records for transformation into useful knowledge is another challenge [20].

The PHR works as a platform for patients’ and health care providers’ use, enabling the exchange of information with health care systems [21]. PHR has also emerged as a mechanism for patients to make appointments with their health care providers. The aim is to address patients’ evolving needs by using specific methods to improve their care and foresee health issues. The technologies used to process health-related data include machine learning, pattern recognition, applied mathematics, statistics, expert systems, data sharing, and artificial intelligence algorithms [22-24]. Moreover, advances in information and communication technology (ICT) have allowed both the storage and easy access of large amounts of data, allowing the release of physical space, facilitating research and the correlation of data within hospitals. However, the increasing number of patients who need care, especially with the increased life expectancy of people in several countries, has been an obstacle to managing huge databases of medical records.

The health community is constantly facing global epidemics and issues that transcend countries, such as cancer, influenza, AIDS, diabetes, and obesity. Patients who migrate or travel from one country to another could make use of their own PHR to obtain faster and more efficient health services. With the increase in the adoption of wireless technology and mobile devices, this creates opportunities to deliver health care services to patients through a world-standard PHR, although many challenges remain in achieving these benefits [25].

### Electronic Health Records

The EHR, also called the electronic medical record, refers to a structure in digital format of patients’ health data that is maintained throughout their life and is stored accurately in a repository [2]. Health care providers use EHRs, whose data can vary greatly and can include vital signs (such as body temperature, pulse, respiration, and blood pressure), age, weight, medications, allergies, medical examination results, and radiology images that are used to diagnose conditions [2,4]. The EHR is used to support health care professionals and health organizations (eg, hospitals, laboratories, or clinics) for the improved management of patient health data [26]. However, these health records are usually not stored with the same structure in different health organizations. These factors hinder the interoperability of health information among hospitals, clinics, and laboratories [27]. To address some of these problems, the PHR concept was proposed in 2006 [6] and was defined as an ISO (International Organization for Standardization) standard (ISO/TR 14292) in 2012 [10].

### Personal Health Records

**Figure 1:**
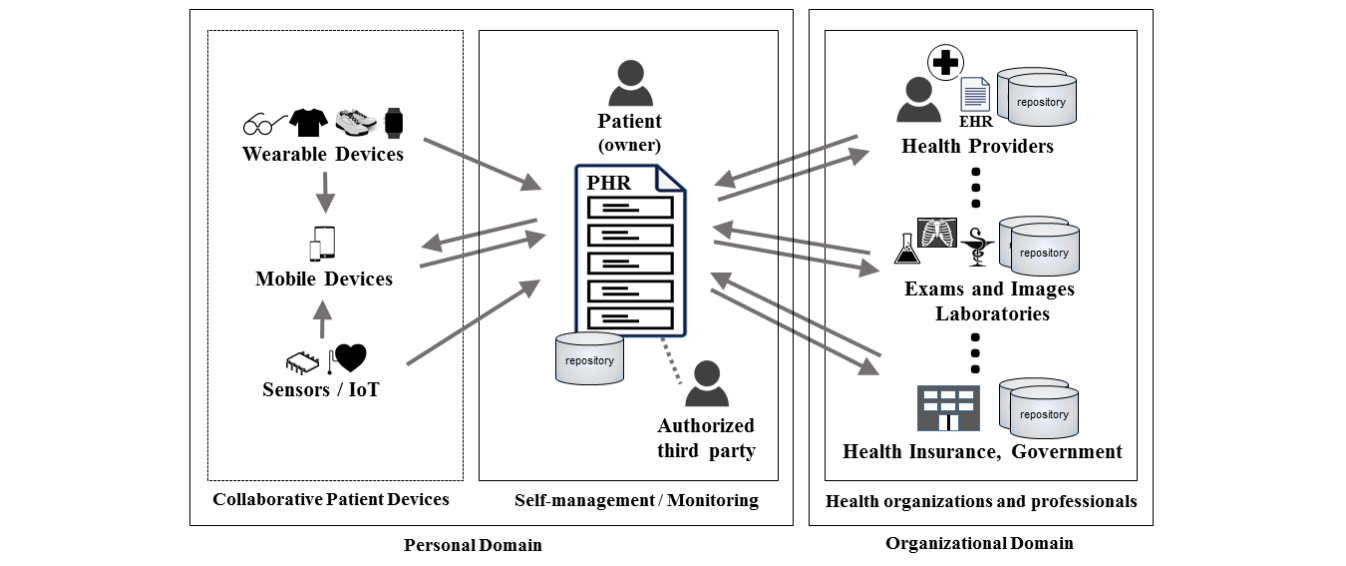
Personal health record (PHR) and electronic health record (EHR) relationships. IoT: Internet of Things.

The PHR refers to a representation of health records related to the care of a patient that is managed by the patient [6]. In other words, the PHR refers to archives containing health data about each patient, but, unlike the EHR, it is managed by the patient [1,10]. With a PHR, patients can choose to share their health data with health care providers or keep them private [6]. Figure 1 illustrates how the PHR and EHR differ in their goals, although they can be integrated to exchange information that is relevant to the patient's health [10].

Multiple EHRs for the same patient can coexist, but only one PHR would exist. The PHR can integrate data from many sources, ranging from devices connected to the patient to health data from EHRs stored in health care provider systems [6].

Although the term PHR may refer to records regardless of format (and can be on paper), the records are implemented electronically and are accessible through mobile devices (mHealth). In this sense, PHRs have allowed patients to self-monitor and manage their own health conditions [23]. Another alternative is medical-oriented PHR, which includes features that are not patient-centered [11,28]. This PHR can be “tethered” (tied) to where the data subsets are provided, including organizations that maintain patient data electronically [6]. In this case, PHRs may be stored in a stand-alone computer or service portal to which only the user has access [29].

Some variant names for PHR appeared in the literature, such as ePHR (electronic PHR) [7] or UHR (universal health record) [30]. The first concept refers to the use of PHR in an electronic format, while the second proposes PHR-sharing data with health care providers. Another term is intelligent PHR (iPHR), which uses medical knowledge to anticipate the health needs of patients and promote tools to guide searches for diseases and recommendations for nursing activities or medical products [19]. Although these different nomenclatures are used, we use the term PHR throughout this work.

To identify the technology for the PHR and to discuss the main open issues, this work surveyed the main contributions of the scientific community over the last decade. The purpose was to review the PHR literature and describe the existing models. As a way of mapping this scenario, we used the systematic literature review methodology to choose the studies [31-33]. As a result, we propose an updated and wide taxonomy for PHRs and indicate further directions for study.

## Methods

### Study Design

This section focuses on describing the study protocol, which introduces the adopted procedures and outlines the main subsequent decisions. As previously mentioned, this study presents a systematic literature review designed to provide a wide overview of the PHR research area, establish whether research evidence exists on a topic, and provide quantitative evidence [31,34]. We selected this type of literature review approach because our goal was to summarize the technology regarding PHRs and identify promising directions, which do not require an in-depth analysis and synthesis. With this in mind, we followed widely recognized empirical guidelines [31,34] to plan and run systematic mapping studies. Moreover, to mitigate threats to validity, we followed the well-documented study protocol available in the studies by Biolchini et al [35] and Qiu et al [36].

The presented systematic literature review method was carried out by defining the following activities:

1. Research questions—introduce the research questions investigated;

2. Search strategy—outline the strategy and libraries explored to collect data;

3. Article selection—explain the criteria for selecting the studies;

4. Distribution of studies—present how studies are distributed chronologically;

5. Quality assessment—describe the quality assessment of the selected studies;

6. Data extraction—compare the selected studies and research questions.

The following sections describe how this process of mapping the study was carried out.

### Research Questions

According to Kitchenham and Charters [31] and Petticrew and Roberts [34], the definition of research questions is the most important part of any systematic review. Therefore, we seek to identify and classify the technology related to PHRs; the features, problems, challenges, and solutions that are currently being considered; and the research opportunities that exist or are emerging. In this sense, we have defined general and specific research questions. The general research questions have been refined into more specific questions to better provide a thorough classification and thematic analysis, as well as to pinpoint promising research directions for further investigation. Our research questions are classified into two categories: general question (GQ) and specific question (SQ). Table 1 lists all the research questions investigated.

The GQ group of research questions concerns a broader classification and some challenges concerning PHRs. GQ1 refers to the question of classifying and defining the taxonomy for PHRs. This research question focuses on the interoperability capacity that a PHR can have. This question highlights integration issues of a PHR that is created and maintained by systems that are developed using heterogeneous technologies. GQ2 refers to the key challenges and issues in using PHRs. This is the main factor that will serve as a direct influence in the PHR survey. The purpose is to identify the types of issues that have been raised in the literature in the last decade. The research focuses on identifying the main problems affecting the spread of PHR adoption by patients and health care providers. For this question, we are able to reason with regard to the issues and factors that consequently influence PHR adoption.

With the general research questions, we have also explored some derived specific research questions (SQ group) to improve the study filtering process. These questions have been proposed to pinpoint questions surrounding the adoption of the PHR. SQ1 seeks to identify the data types that a PHR can contain. SQ2 investigates the types and profiles of users who interact with a PHR. SQ3 examines the types of standards that are used in PHR implementations. SQ4 seeks to show the interaction types that a patient has with a PHR. SQ5 concentrates on evaluating the techniques or methods used to input data into a PHR. SQ6 investigates the purposes of a PHR. Finally, SQ7 concentrates on the types and models of PHR architecture.

**Table 1:** Research questions.

Group and identifier		Issue
**General questions (GQ)**		
	GQ1	How would the taxonomy for PHR^a^ classification appear?
	GQ2	What are the challenges and open questions related to PHRs?
**Specific questions (SQ)**		
	SQ1	What are the data types that are included in a PHR?
	SQ2	What are the standards that apply to PHRs?
	SQ3	What are the user types and profiles that interact with a PHR?
	SQ4	What are the interaction types of a patient with a PHR?
	SQ5	Which are the techniques or methods used to input information into a PHR?
	SQ6	What are the goals of a PHR?
	SQ7	What are the types or models of architecture of PHRs?

^a^PHR: personal health record.

### Search Strategy

The next step was to find a complete set of studies related to the research questions. This process involved the designation of *search keywords* and the *definition of search scope* [34]. In the *construction of search keywords* phase, we defined keywords to obtain accurate search results. In their report, Kitchenham and Charters [31] suggest breaking down the research question into individual facets as research units, where their synonyms, acronyms, abbreviations, and alternative spellings are all included and combined by Boolean operators. In addition, Petticrew and Roberts [34] propose the PICOC (population, intervention, comparison, outcome, and context) criteria, which can be seen as guidelines to properly define such research units.

In focusing on defining the PHR technology, we defined broader PICOC criteria based on the general research questions. Our goal was to refine and answer the specific research questions, which are derived from the general research questions with a restricted focus. Therefore, under the PHR scenarios, we defined the PICOC criteria as follows.

#### Population

The populations involve keywords, related terms, variants, or the same meaning for the technologies and standards on PHRs. Therefore, the following search string in Textbox 1 was defined for the selection.

#### Intervention

We used the following terms to better filter studies in line with the purposes: health data, health services monitoring and reporting, patient monitoring devices, remote health monitoring, and mobile health care devices.

#### Comparison

This case refers to the comparison of different architecture types and models of implementation of the PHR. In addition, we compared the different PHR types regarding coverage and localization.

#### Outcome

The outcomes related to factors of importance to practitioners (eg, improved reliability) and, in particular, to the patient. With respect to PHRs, this might refer to reducing the cost of collecting data, improving health information quality, anticipating potential problems, and allowing the patients to interact with their health data.

#### Context

In this regard, we analyzed the context of PHR information coverage in terms of content such as standardization, information grouping, and security and privacy in the relationships between patients and health care providers.

Hence, the final keyword set is displayed in Textbox 2.

**Search string. PHR: personal health record; PHA: personal health application; PHM: personal health management; PHI: private health information.**

(((“personal” or “patient” or “private”) and (“health”) and (“record” or “application” or “management” or “information”)) or (“patient” and (“access” or “portal”)) or (“PHR” or “PHA” or “PHM” or “PHI”))

**Final keyword set.**

Keywords = PICOC = Population AND Intervention AND Comparison AND Outcome AND Context

In the *definition of search scope* phase, the source studies were obtained from selected electronic databases by searching using the constructed research keywords.

### Article Selection

Once we found all the related articles, we proceeded to remove the studies that were not as relevant and kept only those that were the most representative. Therefore, we removed the studies that did not address PHR specifically. To apply the exclusion criteria, we used the terms of population and intervention criteria as follows:

- Exclusion criterion 1: article does not address PHR or related acronyms (population criterion I).
- Exclusion criterion 2: article does not address “health data” or “health services” (intervention criterion II).

The steps of the filtering process are as follows: (1) impurity removal, (2) filter by title and abstract, (3) removal of duplicates, and (4) filter by full text.

First, the impurities of the search results were removed. Some impurities, for example, the names of conferences correlated to the search keywords, were included in the search results because of the characteristics of the different electronic databases.

Second, we analyzed the title and abstract of the articles and excluded those that did not address PHR as a subject.

Third, all the remaining studies were grouped and the duplicates were removed because some studies were in more than one database.

Some studies remained that were not particularly related to this survey. We analyzed the full text to remove those that were not relevant.

### Quality Assessment

Since it is important and essential to assess the quality of the selected studies, the quality criterion is intended to verify that the article is really a relevant study [31]. We evaluated the selected articles with regard to the purpose of research, contextualization, literature review, related work, methodology, the results obtained, and the conclusion in accordance with objectives and indication of future studies. For this purpose, the quality was evaluated according to Table 2, where the questions to which the articles were submitted to validate that these studies met the quality criteria are listed.

### Data Extraction

We also developed an evaluation form for the selected articles in order to gather information about the studies and the sections where we found answers to general and specific research questions, which are presented in Table 3. This table shows each item of the study related to the research question, allowing us to assess and extract details of the articles and understand how the studies have addressed the issues related to the proposed research questions. The aim was to direct the survey to specific points that would answer the research questions.

**Table 2:** Quality assessment criteria.

Identifier	Issue
C1	Does the article clearly show the purpose of the research?
C2	Does the article adequately describe the literature review, background, or context?
C3	Does the article present the related work with regard to the main contribution?
C4	Does the article have an architecture proposal or research methodology described?
C5	Does the article have research results?
C6	Does the article present a conclusion related to the research objectives?
C7	Does the article recommend future works, improvements, or further studies?

**Table 3:** Review articles related to the research questions.

Section		Description	Research questions
**Open content**			
	Title	Title of the scientific article	GQ1^a^, GQ2, SQ1^b^, SQ2, SQ7
	Abstract	Summary of paper’s purpose, method, and results	GQ1, GQ2, SQ1, SQ2, SQ7
	Keywords	Words representing the text content	GQ1, GQ2, SQ1, SQ2, SQ7
**Article content**			
	Introduction	Introduction specifies the issue to be addressed	All questions
	Background	Section includes concepts and is related to the proposal	All questions
	Method	Presents and describes the scientific methodology	All questions
	Results	Performs an evaluation according to the proposed methodology	All questions
	Discussion	Data that were quantified compared with the literature	GQ2, SQ2-SQ7
	Conclusion	Findings related to the objectives and hypotheses	GQ2, SQ2-SQ7

^a^GQ: general question.

^b^SQ: specific question.

## Results

### Recruitment

In this section, we present the results obtained from the 48 fully assessed studies related to the research topic. We seek to answer each proposed research question in the following subsections through elaborative information synthesis. As a result, aside from answering the research questions, we have also proposed contributions in the PHR field from the study of related works, which are an updated taxonomy and an updated vision about main challenges and issues, as well as an updated survey about data types, standards, user types, profiles, and input techniques.

### Conducting the Search Strategy

To cover as many related studies as possible, we selected 12 electronic databases as our search scope, which are listed in Multimedia Appendix 1. These portals cover the most relevant journals and conferences within the computer science and health care field. In Multimedia Appendix 2, we present the publishers or organization editors and the respective publications of the selected studies. Duplicated results produced from different databases were excluded by manual filtering in the study selection. To limit our search, we set the years to range from 2006 to 2016.

### Proceeding With Article Selection

**Figure 2:**
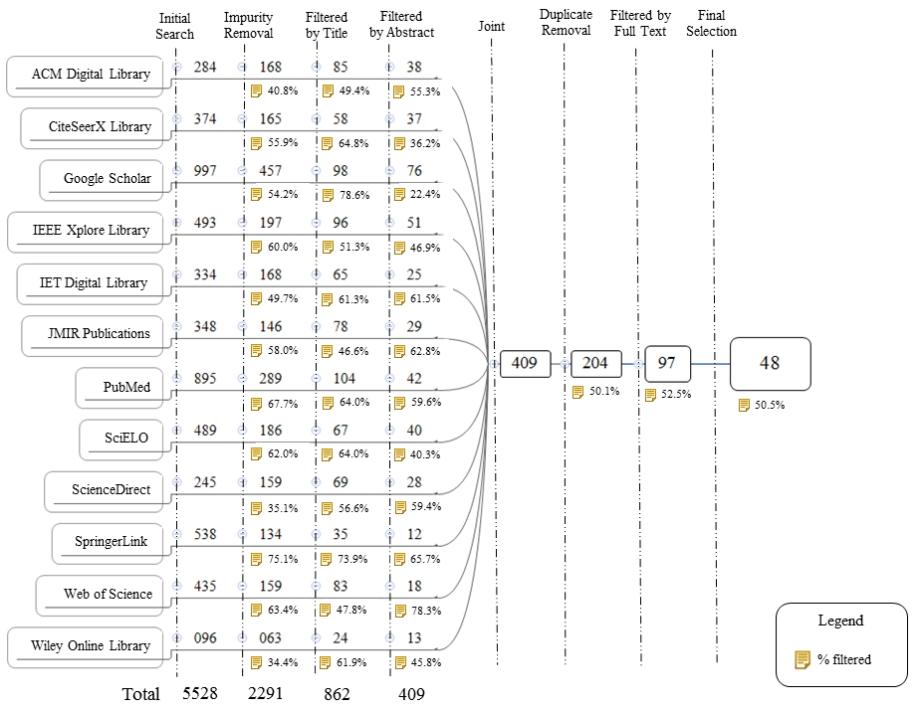
Systematic mapping study—article selection. SciELO: Scientific Electronic Library Online.

The selection process is summarized in Figure 2, which shows the filtering process.

We found 5528 articles in the initial search before applying the exclusion criteria; of these, 3237 (58.55%) articles were identified as impurities. We applied the first exclusion criterion to the studies that remained after we withdrew these articles. Continuing the process, 1429/2291 (62.37%) articles were filtered through a title review, and 453/862 (52.5%) articles were filtered through abstract analysis. We grouped the studies that remained, and 205/409 (50.1%) articles were identified as duplicates and were removed. After this stage, exclusion criterion 2 was applied to the full text and only 97/204 (47.5%) remained.

When analyzing the 97 candidate articles in the list, we noticed that some of these studies were from the same author or research group and were similar in many respects. Some of these articles had been more recent or were even more complete versions but they remained essentially the same methods and techniques. For articles that were repeated, the most representative article was selected. Thus, 49 (50%, 49/97) articles were excluded at this stage. Finally, 48 articles were selected as the baseline for the study. An overview of all primary studies is presented in Table 4 with the identifier, reference, publication year, publisher, and type, which are sorted in ascending order by publication year.

**Table 4:** List of articles.

Identifier	Study, year	Publisher	Type
A01	Bricon-Souf and Newman, 2006 [37]	Elsevier	Journal
A02	Tang et al, 2006 [6]	Oxford^a^	Journal
A03	Frost and Massagli, 2008 [38]	JMIR^b^	Journal
A04	Kaelber et al, 2008 [39]	Oxford	Journal
A05	Huda et al, 2009 [40]	IEEE^c^	Conference
A06	Kim et al, 2009 [41]	JMIR	Journal
A07	Brennan et al, 2010 [3]	Elsevier	Journal
A08	Castillo et al, 2010 [5]	BioMed^d^	Journal
A09	Horan et al, 2010 [23]	JMIR	Journal
A10	Hudson and Cohen, 2010 [22]	IEEE	Conference
A11	Jones et al, 2010 [42]	MLA^e^	Journal
A12	Nazi et al, 2010 [43]	Springer	Journal
A13	Patel et al, 2010 [44]	Elsevier	Journal
A14	Reti et al, 2010 [45]	Oxford	Journal
A15	Wen et al, 2010 [46]	JMIR	Journal
A16	Williams, 2010 [47]	ACM^f^	Conference
A17	Wynia and Dunn, 2010 [7]	Wiley	Journal
A18	Archer et al, 2011 [29]	Oxford	Journal
A19	Baird et al, 2011 [1]	ACM	Conference
A20	Caligtan and Dykes, 2011 [26]	Elsevier	Conference
A21	Lafky and Horan, 2011 [14]	SAGE	Journal
A22	Liu et al, 2011 [48]	ACM	Conference
A23	Siek et al, 2011 [49]	Springer	Journal
A24	Zulman et al, 2011 [50]	ACP^g^	Journal
A25	Carrión Señor et al, 2012 [51]	JMIR	Journal
A26	Emani et al, 2012 [52]	JMIR	Journal
A27	Fuji et al, 2012 [11]	Springer	Journal
A28	Kharrazi et al, 2012 [53]	Elsevier	Journal
A29	Luo et al, 2012 [19]	Springer	Journal
A30	Steele et al, 2012 [54]	Wiley	Journal
A31	Sunyaev and Chornyi, 2012 [55]	ACM	Journal
A32	Agarwal et al, 2013 [56]	JMIR	Journal
A33	Li et al, 2013 [13]	IEEE	Journal
A34	Nazi, 2013 [57]	JMIR	Journal
A35	Woods et al, 2013 [58]	JMIR	Journal
A36	Ancker et al, 2014 [59]	Springer	Journal
A37	Bouri and Ravi, 2014 [60]	JMIR	Journal
A38	Cahill et al, 2014 [21]	Springer	Journal
A39	Chrischilles et al, 2014 [61]	Oxford	Journal
A40	Ozok et al, 2014 [15]	Elsevier	Journal
A41	Spil and Klein, 2014 [62]	IEEE	Conference
A42	Wells et al, 2014 [25]	Oxford	Journal
A43	Czaja et al, 2015 [63]	SAGE	Journal
A44	Liu et al, 2015 [12]	Elsevier	Journal
A45	Price et al, 2015 [64]	BioMed	Journal
A46	Spil and Klein, 2015 [9]	Elsevier	Journal
A47	Sujansky and Kunz, 2015 [65]	Springer	Journal
A48	Ford et al, 2016 [66]	JMIR	Journal

^a^Oxford: Oxford University Press.

^b^JMIR: JMIR Publications.

^c^IEEE: Institute of Electrical and Electronics Engineers.

^d^BioMed: BioMed Central.

^e^MLA: Medical Library Association.

^f^ACM: Association for Computing Machinery.

^g^ACP: American College of Physicians.

**Figure 3:**
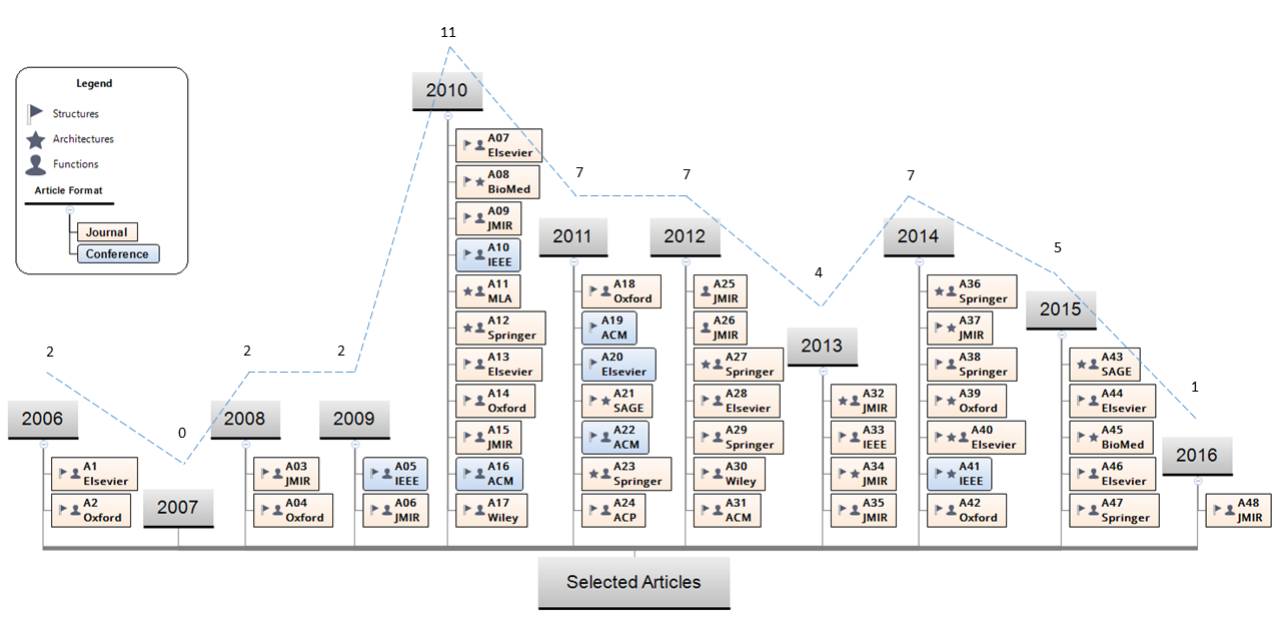
Publication chronology. The numbers above years indicate the number of articles published. Oxford: Oxford University Press; JMIR: JMIR Publications; IEEE: Institute of Electrical and Electronics Engineers; BioMed: BioMed Central; MLA: Medical Library Association; ACM: Association for Computing Machinery; ACP: American College of Physicians.

In Figure 3, we present the evolution of the selected publications over the years, ranging from 2006 to 2016. The studies were analyzed according to the main objectives, as seen in the figure legend, where the articles were divided into the groups “Structures,” “Architectures,” and “Functions.” Above each year, the number of articles published in that year is shown. Each item label includes the publisher of the work, and the journal and conference articles are distinguished by the box format.

### Performing the Quality Assessment

**Figure 4:**
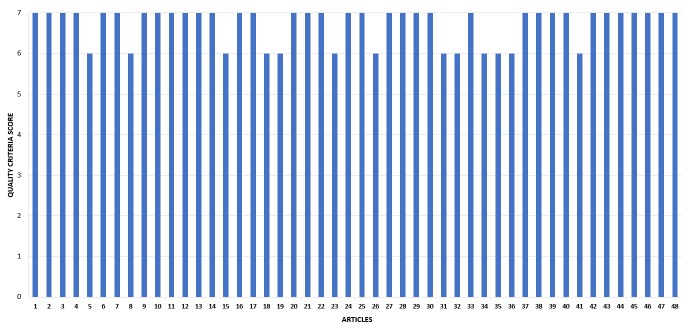
Quality assessment of the articles.

In Figure 4, we present the quality criteria score of the articles based on the quality assessment criteria proposed in Table 2.

The quality criteria score each article obtained is shown on the vertical axis and the studies themselves on the horizontal axis, from 1 to 48. Upon analysis, most articles met all the criteria for evaluation, responding positively to at least 6 out of 7 quality assessment criteria. For instance, several articles do not comment on or cite possible future studies in general because they are conclusive articles, with a conclusion on its assessment.

### Data Extraction and Answers to the Research Questions

Finally, to address the *general research questions*, we have identified the following.

#### GQ1: How Would the Taxonomy for PHR Classification Appear?

We identified studies that investigated a number of current issues that were addressed in the PHR field. Therefore, we managed to build the proposed taxonomy to gather and organize the various possibilities for PHRs. By analyzing the selected articles and seeking to answer this general research question, we propose a taxonomy for PHR based on important characteristics of the models, and we believe that this taxonomy could help to classify, compare, and evaluate different PHR types. Moreover, this classification can provide an overview of possible alternatives in terms of aims, content, and architectures. The proposed taxonomy for the PHR classification is summarized in Table 5, which is broadly divided into three groups: (1) Structures, (2) Functions, and (3) Architectures. Beside each item in Table 5 is a brief description of each classification. The specific research questions (SQ1 to SQ7) are included in the taxonomy, which was developed through analysis of the selected articles.

#### GQ2: What Are the Challenges and Open Questions Related to PHRs?

To answer this question, we listed and identified challenges, open questions, aspects, issues, and common concerns in the adoption of PHR among the analyzed studies. These aspects were collected and are presented in Table 6. As seen, the content is split to group some of the common characteristics of challenges and concerns (GCC, group of challenges and concerns) related to collaboration and communication (GCC1), privacy, security, and trust (GCC2), infrastructure (GCC3), and integration (GCC4). The subject matter that is most commonly cited is separated by item, with the identifiers ranging from CC01 to CC15.

**Table 5:** Personal health record taxonomy.

Group and item		Description
**Structures**		Main data types and standards used in health records
	Data types	Data types found in PHRs^a^ (see subsection SQ1^b^)
	Standards	Standards to which PHRs can adhere (see subsection SQ2)
**Functions**		Depicts the main goals and features present in the PHRs
	Users profiles	User types and profiles that interact (see subsection SQ3)
	Interaction	Patient’s interaction types with a PHR (see subsection SQ4)
	Data source	Techniques for input of information (see subsection SQ5)
	Goals	Represents the aim of the PHR (see subsection SQ6)
**Architectures**		Architecture types and scopes (see subsection SQ7)
	Models	Describes the main architecture models
	Coverage	Has a physical location division for data

^a^PHR: personal health record

^b^SQ: specific question

**Table 6:** Personal health record challenges and concerns.

Group and identifier		Challenge and concern	Reference articles
**GCC1^a^: collaboration and communication**			
	CC01^b^	Context-aware computing	A01, A41
	CC02	Wearable computing, IoT^c^	A01, A28
	CC03	AI^d^ applied to health	A01, A10, A16
	CC04	Personalization, usability, familiarity, comfort	A02, A07, A19, A22, A29, A40, A42, A45
	CC05	Manage medications	A23, A29
	CC06	Patient-generated data	A22, A42, A44, A45, A47
**GCC2: privacy, security, and trust**			
	CC07	Confidentiality and integrity	A07, A08, A19, A29, A42, A45, A46
	CC08	Data repository ownership	A13, A16, A19, A45, A47
	CC09	Authorization and access control technologies	A02, A07, A11, A16, A21, A22, A31, A40, A42
	CC10	Secure transport protocol	A16, A22, A42, A47
**GCC3: infrastructure**			
	CC11	Portability—devices, equipment, hardware	A11, A18, A21, A23, A24, A28, A30, A42, A43, A44
	CC12	Efficiency and scalability	A01, A40, A41, A44, A45, A46
**GCC4: integration**			
	CC13	Patterns in collecting medical data	A13, A17, A42, A47
	CC14	Terminology	A22, A29
	CC15	Interoperability	A13, A16, A21

^a^GCC: group of challenges and concerns.

^b^CC: challenge and concern.

^c^IoT: Internet of Things.

^d^AI: artificial intelligence.

In GCC1 group, there are challenges and issues related to collaboration and communication, ranging from data types to be stored and made available in the PHR to policy barriers to limit the provided information type. Some articles mention the PHR data that are available according to the context awareness, such as CC01, and some articles discuss wearable computing and IoT, such as CC02. Other articles examine artificial intelligence that is applied to the health sector in CC03. The customization, usability, familiarity, and comfort when using the PHR is the subject matter of several articles in CC04, and the management of medications contained in the PHR is reviewed in CC05. The GCC2 group presents issues related to privacy, security, and reliability that are presented in PHRs: CC07 addresses confidentiality and integrity issues. CC08 refers to data repositories and their owners. CC09 examines access control technologies. CC10 includes a discussion on data transport protocols. The GCC3 group treats issues related to the infrastructure of PHRs, in which CC11 discusses the portability of devices and equipment used with a PHR. In CC12, issues on the efficient construction of computer systems and the scalability of the infrastructure used to support PHR solutions are discussed. Finally, in the GCC4 group, concerns about integration are examined, such as in CC13, which concerns patterns in collecting medical data. CC14 presents concerns about the terminology used to collect and store PHRs. Additionally, CC15 addresses issues about interoperability.

Regarding the *specific research questions*, we have identified the following:

#### SQ1: What Are the Data Types That Are Included in a PHR?

To answer this research question, we analyzed all selected studies that involved research of the data types used in PHRs, which are summarized in Table 7. Through the analysis of proposals and references in selected articles, we were able to obtain an updated set of data types related to PHRs. The data types ranged from information cited in many studies, such as those on allergies, immunizations, and medications, to types that are not frequently mentioned, such as genetic information and home monitoring data.

**Table 7:** Personal health record data types.

Type	Description	Reference articles
Allergies	Allergies and adverse reactions	A02, A12, A16, A18, A20, A25, A28, A30, A35, A39, A40, A41, A46
Demographic	Patient statistics and clinical data	A03, A20, A35, A39, A40, A43
Documents	Attached files (photos, scanned documents)	A07, A20, A28
Evolution	Progress and clinic notes, care plan	A07, A14, A18, A34
Family history	Family medical history	A02, A12, A16, A18, A20, A25, A28, A37
General	Patient registration information, emergency contact	A03, A12, A16, A18, A28
Genetic	Genetic information	A16, A25
Home monitor	Home-monitored data	A02, A18, A25
Immunizations	Immunization records (vaccine), tracking immunizations	A02, A09, A12, A16, A18, A19, A20, A25, A28, A30, A32, A37
Insurance	Insurance plan information, coding for billing	A16, A18, A28
Laboratory results	Laboratory and imaging test results (laboratory tests)	A02, A12, A14, A16, A18, A19, A20, A25, A28, A32, A35, A43
Major illnesses	List of major diseases	A03, A02, A12, A18, A25
Medications	Medication list prescribed, past medicines taken	A02, A07, A12, A16, A18, A20, A25, A28, A35, A39, A41
Prescriptions	Medical prescription refills (renewing)	A04, A09, A12, A15, A17, A43, A46
Prevention	Preventive health recommendations	A12, A18, A32, A40, A46
Providers	Previous health care provider list	A02, A18, A28, A30, A37
Scheduling	Appointments, past procedures, hospitalizations	A02, A12, A16, A18, A20, A25, A28, A35, A37
Social history	Social history, lifestyle (health habits)	A02, A12, A18, A25, A40
Summaries	Admissions, permanencies, and discharges	A39, A35, A43
Vital signs	Status of bodily functions	A16, A30, A35, A37, A40

#### SQ2: What Are the Standards That Apply to PHRs?

Some providers use proprietary formats to organize their health records that are used only by internal applications, each of which has a different format [7,65]. Thus, to answer this question, we focused on open standards, which are summarized in Table 8 and present a vast number of data organizational patterns for health records. Table 8 lists the referenced standards (group of standards, GS) according to their goals: nomenclature and terminology (GS1), privacy (GS2), structural and semantic (GS3), and templates and technology platforms (GS4). In group GS1, standards regarding nomenclature and terminology were grouped. Group GS2 contains only one standard that addresses privacy. In the GS3 group, several structural and semantic standards are presented. Finally, the GS4 group is related to templates and technology platform standards. We were able to identify some standards from the research on integrations and related projects, such as openEHR [67], which is integrated with the DICOM (Digital Imaging and Communications in Medicine) standard and others.

**Table 8:** Main personal health record–related standards.

Group and standard		Description	Reference articles
**GS1^a^: nomenclature and terminology**			
	HNA/NIC^b^	Classifications of nursing activities and interventions	A29
	ICDx	Family of international classification of diseases	A11, A28, A29, A44
	LOINC	Code names for identifying medical observations	A47
	SNOMED CT	Terminology collection of medical terms	A11, A28, A47
	UMLS	System of medical vocabularies	A11, A13
**GS2: privacy**			
	HIPAA	USA legislation for medical information	A09, A22, A25, A35
**GS3: structural and semantic**			
	ASC X12N	Accredited standards committee X12-INS	A45, A47
	CCD	Specification for exchange clinical documents	A11, A47, A48
	CCR	Specification for sharing continuity of care content	A11, A33
	CDA	Specification for clinical notes	A11, A47
	DICOM	Standard for medical digital imaging	A11
	EN 13606	EHR^c^ standards in Europe	A25
	HL7/FHIR/SMART	Family of standards and platforms based on the HL7 reference model	A11, A18, A28, A42, A43, A45, A47
	ISO^d^	TR (Technical Report) 14292 (PHR) and ISO/IEEE 11073 Personal Health Data (PHD)	A01, A03, A20, A23, A25, A38, A43, A47
	openEHR	Open standards specification in eHealth	A11
	xDT	German family of data exchange formats	A04
**GS4: templates and technology platforms**			
	OpenMRS	Platform and reference application named Open Medical Record System	A42
	OSCAR	EHR system named Open Source Clinical Application and Resource	A42

^a^GS: group of standards.

^b^HNA/NIC: Home Nursing Activities/Nursing Interventions Classification

^c^EHR: electronic health record.

^d^ISO: International Organization for Standardization.

#### SQ3: What Are the User Types and Profiles That Interact With a PHR?

Upon analyzing the selected articles, we identified a set of profiles or user types that have access to the electronic patient record, which vary from the physician, who is primarily responsible for the PHR information, to the patient. The types of access also include the possibility that some data may be publicly available, for example, on social networks [19]. There are multiple stakeholders involved in accessing the PHR, such as patients, providers, employers, payers, governments, and research institutions [6]. In Multimedia Appendix 3, we present the details of the profiles that have been identified. We can see that the physician is widely referenced, while the nurse and administrative profiles are not cited as often. Among the laity, the patient profile is often cited; however, the relative or guardian profile is less commonly cited. We also included a public profile because patients might share their information anonymously in some cases or for other cases in which public administration sectors provide open statistical data.

In the following section, we present a brief description of the perceived profiles:

Physician or doctor—the physician, in this assessment, is the health professional profile responsible for reporting patient data in consumer electronic records.

Nurse—according to the International Standard Classification of Occupations [68], nursing professionals provide treatment, support, and care for people who need nursing care owing to the effects of aging, injury, disease, or other physical or mental impairments or face potential risks to their health.

Administrative—this profile refers to all administrative health professionals who are not directly linked to the data generation but have informational access for bureaucratic, statistical data gathering or financial information needs.

Patient or consumer—this profile refers to the PHR principles; some authors also refer to the patient as a consumer of health care [14,26].

Relative—this profile is composed of parents, guardians, caregivers, responsible legal individuals, or anyone who has the patient’s permission to access his or her PHR.

Public or anonymous—this refers to profiles with external access in an anonymous or public way, such as institutions, the government, researchers, health plans, third parties, and even social networks.

#### SQ4: What Are the Interaction Types of a Patient With a PHR?

This research question seeks to describe the interaction types of a patient with a PHR, that is, the types of relationships that a patient has using the PHR. In the following section, we present a brief description of the interaction types that were identified when analyzing the articles:

Direct—in this case, the patients are the owners and manage their health data in the PHR. Reference articles: A02, A05, A09, A12, A25, A26, A31, A48.

Indirect—in this case, the patient has read-only access and cannot edit the data. The health care providers are the owners, and the patient can only download or print the health records. Reference articles: A01, A05, A22, A25, A26, A40, A41, A42.

Outsourced—in this case, the patient authorizes a third party to handle the health data or the responsible parties (eg, parents) manage the patient's health records. Reference articles: A02, A03, A04, A07, A18, A24, A25, A28, A37, A48.

#### SQ5: Which Are the Techniques or Methods Used to Input Information Into a PHR?

Another result was the identification of techniques and actors that interact in the process of data collection for inputting into a PHR. Table 9 presents some answers to this specific research question, summarizing the techniques of inputting the relevant data into PHRs.

**Table 9:** Techniques for inputting information into personal health records.

Techniques and profiles (actors)		Description	Reference articles
**Data collaboration (T1^a^)**			
	Health professionals	Collaboration between multiple health care professionals. Health care providers are the owners (paternalistic relationship).	A08, A09, A12, A15, A22, A23
**Patient reports (T2)**			
	Patient	Patient reports data, for example, listing drugs that are being used or menstrual period data.	A23, A26, A47
**Adaptive platforms (T3)**			
	Environment	Aggregate sources provisioning individualized personal eHealth services combined with context information, including monitoring sensors. Patient and health care providers collaborate for inputting data into PHR^b^.	A01, A26, A38, A43, A44
**Anonymization (T4)**			
	Anonymous	Anonymizing social network data.	A16, A44

^a^T: technique.

^b^PHR: personal health record.

This information follows standards and is intended to structure and standardize the data provided. We list the main actors that provide the data, including health professionals and the patients themselves, which are gathered from the environment, including anonymously. The techniques (T) identified for inputting data range from data collaboration (T1), to patient reports (T2), adaptive platforms (T3), and anonymization (T4). Table 9 also includes articles in which these techniques and actors are cited. In short, this was the actors’ group that was identified with a relevant interaction in collecting data for inputting data into the PHR.

#### SQ6: What Are the Goals of a PHR?

This research question includes the main goals of the PHR. This question is intended to identify the purpose that a PHR has in a broad context and that applies to any profile that has access. In the following section, we present a brief description of the interaction types:

Consult—in this case, the purpose is to allow the profile to only consult (in read-only mode). Reference articles: A01, A03, A07, A10, A13, A15, A16, A17, A21, A39, A47.

Maintain—in this case, the user profile is allowed to maintain and control the health records. Reference articles: A09, A16, A18, A22, A29, A33, A37, A46.

Monitor—in this case, the PHR is in monitoring mode and can send alerts or warnings for one or more profiles; the goal is to help the patients monitor their health. Reference articles: A01, A07, A10, A20, A23, A25, A29, A40, A43, A45.

#### SQ7: What Are the Types or Models of Architecture of PHRs?

The purpose of this question is to identify the types or models of architecture in which a PHR can be implemented. When analyzing the articles, as seen in Table 10, the architecture types (architecture group, AG) were split into two groups: model (AG1) and coverage (AG2). The first group, AG1, describes the main architecture models. The second group, AG2, divides the data based on the physical location, that is, the scope of the PHR.

**Table 10:** Personal health record architecture types or models.

Group and item		Description	Reference articles
**AG1^a^: model**			
	On paper	Health records are kept on paper	A08, A20, A22
	Inside	PHR^b^ is kept in local repositories, inside the provider, for example	A02, A03, A16, A20, A31
	Outside	PHR is distributed or shared between servers outside the provider	A01, A03, A24, A35
	Hybrid	PHR is distributed inside and outside the provider	A02, A10, A28, A35, A47
**AG2: coverage**			
	Stand-alone	Data coverage is used only in the provider area	A11, A26, A45, A46
	Local	Area is at the city level	A03, A11, A20, A29, A35
	Regional	Data are used in the state or province	A02, A04, A25, A37, A45
	National	Coverage encompasses the nation	A09, A12, A28, A34, A35
	International	Coverage transcends the nation	A09, A16, A28, A30

^a^AG: architecture group.

^b^PHR: personal health record.

## Discussion

### Principal Findings

In this study, we sought to identify a quantitative and qualitative sample of studies that enabled us to obtain a clear overview of the technology regarding PHRs in the last 10 years from a number of candidate articles. This research sought to highlight some of the most relevant studies of the field according to certain systematic selection criteria. The survey sought to identify several common aspects of studies by answering a number of research questions. As a result, we were able to propose a PHR taxonomy and identify gaps to be further researched that represent challenges and issues that have been detected in recent years. These aspects range from patients’ concerns to providers’ problems regarding PHR adoption. In addition, we have identified the data types included in PHRs, an updated tabulation of the data standardization, access profiles and their characteristics, and, finally, a classification of input techniques. We also identified other common and related aspects. These opportunities are discussed as follows.

#### GQ1: How Would the Taxonomy for PHR Classification Appear?

For the *GQ1 research question*, we sought to define a PHR taxonomy, which is presented in Table 5. Our proposed taxonomy illustrates the PHR types and their organization according to several studies that were analyzed. We primarily identified three major groups of PHR organization types: (1) Structures, (2) Functions, and (3) Architectures. From these groups, we were able to examine the PHR types in depth to understand each one of them. These groups also showed that there are PHR application initiatives on several fronts with concerns that range from features and content to architectural format in terms of PHR implementation [54].

#### GQ2: What Are the Challenges and Open Questions Related to PHRs?

For the *GQ2 research question*, we sought to define the main challenges and issues regarding the use of PHRs. There are many open questions to be further researched in the area of PHR. The challenges and constraints in the adoption of PHRs are diverse. Some research results indicate problems of usability, privacy, security, and complexity in the use of PHRs, ranging from fears of including erroneous data to the difficulty of interpretation as the main difficulties [1,48]. In Table 6, we describe some challenges and issues that may give rise to future studies. According to the number of items in each group in the table, we notice a greater concern with the first three groups, although we cannot claim this assessment as being definitive. One possibility that we touch upon for this observation is that the integration of standards and interoperability, as well as the nomenclatures and terminologies, are already in a stage of stability and consolidation. This leads us to reinforce the thesis that the concerns of the authors at this time are the issues raised by the first three groups of problems. That is, the concerns and challenges are more focused on discussions regarding confidentiality, integrity, authorization, access control, portability, efficiency, scalability of solutions, and issues related to user experience.

#### SQ1: What Are the Data Types That Are Included in a PHR?

With respect to the *SQ1 research question*, we sought to define an updated ranking on data types in PHRs. Upon analyzing the studies, we observed that PHR data types have evolved since the first PHRs [6,37]. The data types found include groups that are not usually included in EHRs. Among the EHR stored data are medications, prescriptions, scheduled appointments, vital signs, medical history, laboratory information, immunizations, summaries, scanned documents, billing information, and progress notes about changes in the patient's health [4]. However, in PHRs, new data types have emerged, including genetic information [47,51], medical advice (recommendations), and prevention concerning the patient's health, as well as data types with recommendations for prevention and home monitoring data [9,15]. Other data types that appear in PHRs are allergies, patient registration data, and insurance plan information, including demographic data such as age, sex, and education. Furthermore, information on the patient’s family, social history, lifestyle, food, diet, daily activities, and a list of providers who treated the patient previously are included in PHRs.

#### SQ2: What Are the Standards That Apply to PHRs?

For the *SQ2 research question*, we sought to define a current view of PHR standards. The result was the identification of the current list of existing data standards used in PHRs. We observed several standards that were maintained by various stakeholders that were located in different countries and regions. We were also able to observe a consolidation of some patterns in the articles’ citations, such as ISO [4,10] and HL7 (Health Level Seven) [29], which are used to define and establish interoperability between the systems. When analyzing the articles, it was observed that all the standards listed can be used directly or indirectly with a PHR. However, their purposes are diverse. Some standards have specific goals, for example, DICOM [42] and SNOMED CT [65], while others have broader purposes, for example, HL7 [29] and openEHR [67], which can be integrated with other specific standards to render the solution. Finally, we identified some open systems or platforms that serve as templates, which use some of the listed standards to propose management solutions for patients’ health data.

#### SQ3: What Are the User Types and Profiles That Interact With a PHR?

In the *SQ3 research question*, we sought to define the PHR user types and profiles that address PHR. The result was the identification of updated profiles as well as their characteristics. For the security and privacy of the health data, the answer to this research question offered a clear definition of the profiles that are allowed access to the PHR and what their responsibilities are [11]. In terms of access profiles, although the PHR is focused on personal use, the idea is that a patient can also delegate access to third parties by choice or necessity, as in the case of children or people who need special care. These third parties can access all or only specific parts of the PHR dataset. Patients can share their PHR for various purposes. Such patients may be minors whose parents need to share their health data with physicians, people with special needs who require constant monitoring, or even patients who wish to share their health data with other physicians. By analyzing the selected articles, it was possible to find multiple profiles that have access to the PHR. We can therefore highlight the following profiles: patients, physicians, nurses, relatives, administrators, and the public. A physician’s tasks include recording the health information and medical history of the patients as well as exchanging information with practitioners and other health care professionals [68].

In cases where patients need emergency care, a primary care physician usually treats them. If more specialized care is needed, the physician indicates the need for a specialist. Furthermore, physicians must report births, deaths, and notifiable diseases to the government. Because the PHR is composed of health data that are stored for a lifetime, many physicians edit the PHR over time. Otherwise, in the case of an administrative profile, these professionals usually have limited and controlled access to the medical records. This profile is considered internal access, which is not to be confused with external access institutions. With the patient profile, the user can manage the information provided in his or her repository. The purpose is for patients to have access to their health data and use them throughout their lives [65]. This set of information is established at different moments over time, for example, for each medical consultation, laboratory test, and hospital admission. Nevertheless, there is a clear distinction between what was reported by health professionals and what the patient reports. Thus, the PHR offers an exact distinction between what was reported from each profile in its repository. In the case of a relative profile, some authors distinguish these profiles in terms of accessing the PHR with some limitations or full access with the permission of the patient [5,23,65]. Additionally, in the case of public or anonymous profiles, the health data can be accessed in a limited or shared way, in which the PHR has a public and social nature to help other patients [47].

#### SQ4: What Are the Interaction Types of a Patient With a PHR?

In the *SQ4 research question*, we were able to identify three types of patient interactions with the PHR. In the first type, according to the definition of the PHR in ISO 14292 [10], the patient manages and controls the health data directly. In the second case, the patient only acts in a supporting role as a complementation of EHRs but does not have effective control. Finally, in the third type the patient outsources the management of the health data to a responsible person.

#### SQ5: Which Are the Techniques or Methods Used to Input Information Into a PHR?

Regarding the *SQ5 research question*, we sought to define the main techniques to input data into the PHR. As a result, with the analysis of the selected articles presented in Table 9, we can identify the techniques and profiles of the actors who use them. In the data collaboration (T1) technique, different health professionals access the PHR aside from the patient. The patient remains the PHR owner, but health professionals collaborate on input records in an identifiable and controlled way. In the second case, patient reports (T2), patients alone are in charge of inputting their medical record data without any support. In the third form, adaptive platforms (T3), the reported data and the data collected from the EHR are integrated with the PHR data. In this case, data obtained from different sources and contexts are combined. The purpose is to provide better management of the patient's condition. For instance, it would be possible to provide real-time access to sensitive patient information and ease communication among patients and providers. In the case of the anonymization (T4) technique, medical data can be integrated with a social network, where the patient can share his or her status anonymously and receive contributions from other users.

#### SQ6: What Are the Goals of a PHR?

In the *SQ6 research question*, we sought to identify the PHR use purposes. This research question is related to the specific question SQ3, which aims to identify the objectives of the user profiles when accessing the PHR. We have identified three objective types. In the first case, the user profile accesses the PHR to only verify the health data without manipulating them. One example here includes health professionals or administrators who have permission to only view the data. In the second case, the user profile has permission to manipulate the data. In this situation, it is important to highlight the need to identify and control the profile that has changed the data and which data have been changed. In the third case, the user profile only monitors the records. An example of this might be a case in which the PHR receives data from sensors (IoT) and can send alerts depending on a situation.

#### SQ7: What Are the Types or Models of Architecture of PHRs?

Finally, in the *SQ7 research question*, we identified the architectures related to PHRs. We divided them into two groups: types (AG1) and coverage areas (AG2), as seen in Table 10. In the case of architecture models, some articles state that health data are still stored on paper in many places, and other institutions have evolved into the proposed hybrid architectures with the PHR distributed inside and outside the health care organizations. In the case of the possibilities of coverage areas, we identified types ranging from a stand-alone PHR on a single machine to PHRs that can be taken from one country to another following an open international standard.

### Limitations

This research is limited to aspects related only to PHRs rather than also including EHRs or electronic medical records, for example. In this sense, the review focused exclusively on articles addressing the inherent PHR concepts. This research sought to answer the research questions that were proposed in order to obtain an outline of the current literature related to PHRs without specifically assessing any computer system that refers to the use of PHR. The research was limited to obtaining articles published in a number of scientific portals related to ICT and health. Our research was reduced to studies found from these websites when we implemented the steps of the systematic literature review methodology. We focused our work on scientific articles and did not address commercial or more technological approach solutions.

### Conclusions

This study aimed to raise and discuss the main issues regarding PHRs and identify the concepts of the technology in this area. To answer the research questions in this paper, we sought first to systematize and qualify the information that served as a source for the survey. For the completion of the work, we were able to identify and propose a broad taxonomy for the scope of work, which was created after an analysis of the relevant articles in the last decade. In the taxonomy, we were able to identify and group a number of types and PHR classifications ranging from “Structures” and types associated with “Functions” to the types of “Architectures” applied to PHRs. Having established the taxonomy, it was possible to observe other important relationships to understand PHRs. We noticed aspects regarding concerns and challenges in the adoption of PHRs as well as the main data types. In addition, we were able to identify several standards regarding PHR, where it was possible to verify those that were most important in the current scenario. Regarding user profiles, we identified the main users representing these types of profiles, as well as their responsibilities when they access PHRs. We were able to identify the techniques and methods used in the input of information into PHRs.

Finally, aside from answering all the specific research questions and relating them in the taxonomy, we can also rank the PHR with regard to goals, negotiation types, and architectures. The answers and classifications obtained contribute to the achievement of a coverage degree of searches that are identified in various aspects regarding the PHR. The physician-patient relationship traditionally consists of total dependence of the patient on the physician. In addition, the fragmented nature of the health system can impose a costly burden on physicians. The PHR can be a solution to this problem, although obstacles still persist, including support for reaching this paradigm, where the ownership of the data belongs to the patient.

In future studies, we envision a focus on the challenges and issues related to security, privacy, and trust, which directly affect the users’ confidence in adopting the PHR. Although these questions have existed for a long time, they do not have definitive answers yet. Other aspects that can be studied and that are important to improving the user experience are questions about usability, personalization, familiarity, and comfort. Another aspect that can serve as a future study is to explore the models of architecture and the implementation of PHR following the expansion of the use of technologies such as wearable computing, IoT, and artificial intelligence that are applied to health.

## Appendix

Multimedia Appendix 1 Selected portals.

Multimedia Appendix 2 List of editors.

Multimedia Appendix 3 List of users and profiles access.

Multimedia Appendix 4 Presentation of study.

## Abbreviations

AG: architecture group
DICOM: Digital Imaging and Communications in Medicine
EHR: electronic health record
ePHR: electronic personal health record
GQ: general question
HL7: Health Level Seven
ICT: information and communication technology
IoT: Internet of Things
ISO: International Organization for Standardization
iPHR: intelligent personal health record
GS: group of standards
PHR: personal health record
PICOC: population, intervention, comparison, outcome, and context
SQ: specific question
UHR: universal health record
