# Supplementary material for: Personal Health Records: A Systematic Literature Review
Source: J Med Internet Res. 2017 Jan 6;19(1):e13. doi: 10.2196/jmir.5876 (PMC5251169; doi:10.2196/jmir.5876)
Supplement: Multimedia Appendix 1 [file jmir_v19i1e13_app1.pdf]

Acronym	Portal Name
ACM	ACM Digital Library
CiteSeerX	CiteSeerX Library
Google Scholar	Google Scholar
IEEE	IEEE Xplore Digital Library
IET	IET Digital Library
JMIR	JMIR Publications Library
PubMed	National Center for Biotechnology Information, U.S. National Library of Medicine
SciELO	Scientific Electronic Library Online
ScienceDirect	Elsevier B. V. ScienceDirect
Springer	Springer Science
Web of Science	Web of Science
Wiley	Wiley Online Library
