# Supplementary material for: Personal Health Records: A Systematic Literature Review
Source: J Med Internet Res. 2017 Jan 6;19(1):e13. doi: 10.2196/jmir.5876 (PMC5251169; doi:10.2196/jmir.5876)
Supplement: Multimedia Appendix 2 [file jmir_v19i1e13_app2.pdf]

Publisher and Publications	
**American College of Physicians**	
- Annals of Internal Medicine	
**Association for Computing Machinery**	
- iConference	- Journal of Data and Information Quality
- International Conference on Software Engineering in Health Care	
**BioMed Central**	
- BMC Medical Informatics and Decision Making	
**Elsevier**	
- Applied Ergonomics	- International Journal of Medical Informatics
- Future Generation Computer Systems	- Journal of Biomedical Informatics
- Health Policy and Technology	- Web Semantics
**IEEE**	
- Transactions on Information Technology in Biomedicine	
- Transactions on Parallel and Distributed Systems	
- International Conference on System Sciences	
- International Conference on Pervasive Computing Technologies for Healthcare	
- International Conference of the Engineering in Medicine and Biology	
**JMIR Publications**	
- Journal of Medical Internet Research	
**Medical Library Association**	
- Journal of the Medical Library Association	
**SAGE Publications**	
- Health Informatics Journal	- Human Factors
**Springer Science**	
- Health and Technology	- Journal of Medical Systems
- Journal of General Internal Medicine	- Journal of Neuro-oncology
**The Oxford University Press**	
- Journal of the American Medical Informatics Association	
**Wiley Online Library**	
- The Journal of Law, Medicine & Ethics	
- Journal of the American Society for Information Science and Technology	
