# Supplementary material for: Personal Health Records: A Systematic Literature Review
Source: J Med Internet Res. 2017 Jan 6;19(1):e13. doi: 10.2196/jmir.5876 (PMC5251169; doi:10.2196/jmir.5876)
Supplement: Multimedia Appendix 3 [file jmir_v19i1e13_app3.pdf]

User or profile and articles	Providers and health care professionals			Laity		Public
	Physician, doctor	Nurse	Administrative, other	Patient or consumer	Relatives, caregiver, payer	Government, health plan
A01	✓	✓		✓	✓	✓
A02	✓			✓	✓	✓
A03	✓	✓	✓	✓	✓	✓
A04	✓	✓	✓	✓	✓	✓
A05			✓	✓		✓
A06		✓	✓	✓		✓
A07	✓	✓		✓		✓
A08	✓	✓	✓	✓	✓	✓
A09	✓	✓		✓	✓	✓
A10	✓				✓	✓
A11				✓		✓
A12	✓			✓		✓
A13	✓			✓		✓
A14	✓			✓		✓
A15	✓	✓		✓		✓
A16	✓	✓	✓	✓	✓	✓
A17	✓		✓	✓	✓	✓
A18	✓	✓	✓	✓	✓	✓
A19	✓			✓	✓	✓
A20	✓	✓		✓		✓
A21	✓	✓		✓	✓	✓
A22	✓	✓		✓	✓	✓
A23		✓		✓		✓
A24	✓		✓	✓		✓
A25	✓		✓	✓	✓	✓
A26	✓	✓	✓	✓	✓	
A27	✓			✓		✓
A28	✓	✓		✓	✓	✓
A29	✓	✓		✓	✓	✓
A30	✓			✓	✓	✓
A31	✓			✓	✓	✓
A32				✓	✓	✓
A33	✓	✓		✓	✓	✓
A34	✓	✓	✓	✓		
A35	✓	✓		✓	✓	✓
A36	✓			✓	✓	✓
A37	✓		✓	✓	✓	✓
A38	✓	✓		✓	✓	
A39	✓			✓	✓	
A40	✓			✓		✓
A41	✓			✓	✓	✓
A42	✓			✓	✓	✓
A43	✓			✓		✓
A44	✓	✓	✓	✓	✓	✓
A45	✓		✓	✓	✓	
A46	✓			✓	✓	✓
A47				✓	✓	✓
A48	✓		✓	✓	✓	✓
