# Supplementary material for: Personal Health Records: A Systematic Literature Review
Source: J Med Internet Res. 2017 Jan 6;19(1):e13. doi: 10.2196/jmir.5876 (PMC5251169; doi:10.2196/jmir.5876)
Supplement: Multimedia Appendix 4 [file jmir_v19i1e13_app4.pdf]

Personal Health Records: A Systematic Literature Review

Alex Roehrs, MSc; Cristiano André da Costa, PhD;
Rodrigo da Rosa Righi, PhD; Kleinner Silva Farias de Oliveira, PhD

Programa de Pós-Graduação em Computação Aplicada,
Universidade do Vale do Rio dos Sinos, São Leopoldo, Brazil

DOI:10.2196/jmir.5876

Agenda

Introduction

- PHR and EHR relationships

Method

- Research Questions
- Search Strategy
- Article Selection
- Quality Assessment
- Data Extraction

Results

- Proceeding with Article Selection
- Data Extraction and Answers to the Research Questions

Discussion

- Limitations
- Conclusions

Introduction

- Objective: explore the recent literature related to PHR defining the taxonomy, challenges and open questions
- Methods: using Systematic Literature Review (SLR)
- In addition, to identify:
  - Data types
  - Standards
  - Profiles
  - Goals
  - Methods
  - Functions
  - Architectures

PHR and EHR Relationships

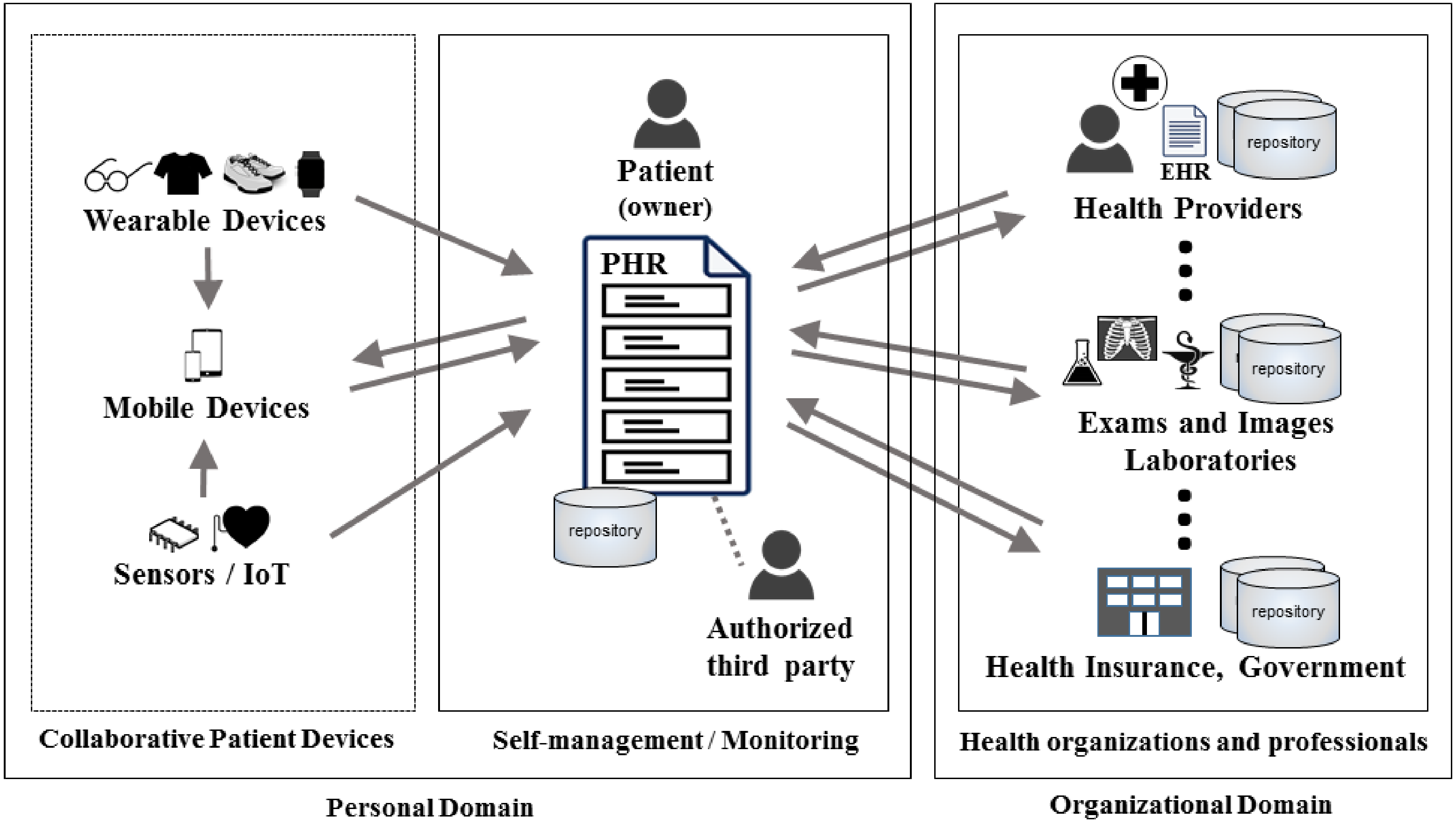

Method

I. Research Questions
II. Search Strategy
III. Article Selection
IV. Quality Assessment
V. Data Extraction

Research Questions

Group / ID	Issue
**General Questions (GQ)**	
GQ1	How would the taxonomy for PHR classification appear?
GQ2	What are the challenges and open questions related to PHRs?
**Specific Questions (SQ)**	
SQ1	What are the data types that are included in a PHR?
SQ2	What are the standards that apply to PHRs?
SQ3	What are the user types and profiles that interact with a PHR?
SQ4	What are the interaction types of a patient with a PHR?
SQ5	Which are the techniques or methods used to input information into a PHR?
SQ6	What are the goals of a PHR?
SQ7	What are the types or models of architectures of PHR?

Search Strategy

- PICOC
  I. **P**opulation
  II. **I**ntervention
  III. **C**omparison
  IV. **O**utcome
  V. **C**ontext

Articles Selection

- Steps
    I. Impurity Removal
    II. Filter by Title and Abstract
    III. Duplicate Removal
    IV. Filter by Full Text

Quality Assessment

ID	Issue
C1	Does the article clearly show the purpose of the research?
C2	Does the article adequately describe the literature review, background or context?
C3	Does the article present the related work with regard to the main contribution?
C4	Does the article have an architecture proposal or research methodology described?
C5	Does the article have research results?
C6	Does the article present a conclusion related to the research objectives?
C7	Does the article recommend future works, improvements or further studies?

Data Extraction

Section	Description	Research Questions
**Open content identification article**		
Title	Title of the scientific article.	GQ1, GQ2, SQ1, SQ2, SQ7
Abstract	Summary of paper's purpose, method and results.	GQ1, GQ2, SQ1, SQ2, SQ7
Keywords	Words representing the text content.	GQ1, GQ2, SQ1, SQ2, SQ7
**Article content**		
Introduction	Introduction gated the issue to be addressed.	All questions.
Background	Section includes concepts and is related to the proposal.	All questions.
Method	Presents and describes the scientific methodology.	All questions.
Results	Performs an evaluation according to the proposed methodology.	All questions.
Discussion	Data that were quantified compared to the literature.	GQ2, SQ2-SQ7
Conclusion	Findings related to the objectives and hypotheses.	GQ2, SQ2-SQ7

Results

- We reviewed more than 5,000 scientific studies published in the last 10 years, selected the most significant approaches, and thoroughly surveyed the healthcare field related to PHRs.
- We obtained an updated taxonomy and identified challenges, open questions, and current data types, related standards, main profiles, input strategies, goals, functions and architectures of PHR.

Proceeding with Article Selection

	Initial Search	Impurity Removal	% filtered	Filtered by Title	% filtered	Filtered by Abstract	% filtered
ACM Digital Library	284	168	41%	85	49%	38	55%
CiteSeerX Library	374	165	56%	58	65%	37	36%
Google Scholar	997	457	54%	98	79%	76	22%
IEEE Xplore Library	493	197	60%	96	51%	51	47%
IET Digital Library	334	168	50%	65	61%	25	62%
JMIR Publications	348	146	58%	78	47%	29	63%
PubMed	895	289	68%	104	64%	42	60%
Scielo	489	186	62%	67	64%	40	40%
ScienceDirect	245	159	35%	69	57%	28	59%
Springer Link	538	134	75%	35	74%	12	66%
Web of Science	435	159	63%	83	48%	18	78%
Wiley Online Library	096	063	34%	24	62%	13	46%
Total	5528	2291		862		409	

Joint	Duplicate Removal	Filtered by Full Text	Final Selection
409	204 (50%)	97 (52%)	48 (51%)

Legend: % filtered

Publication Chronology

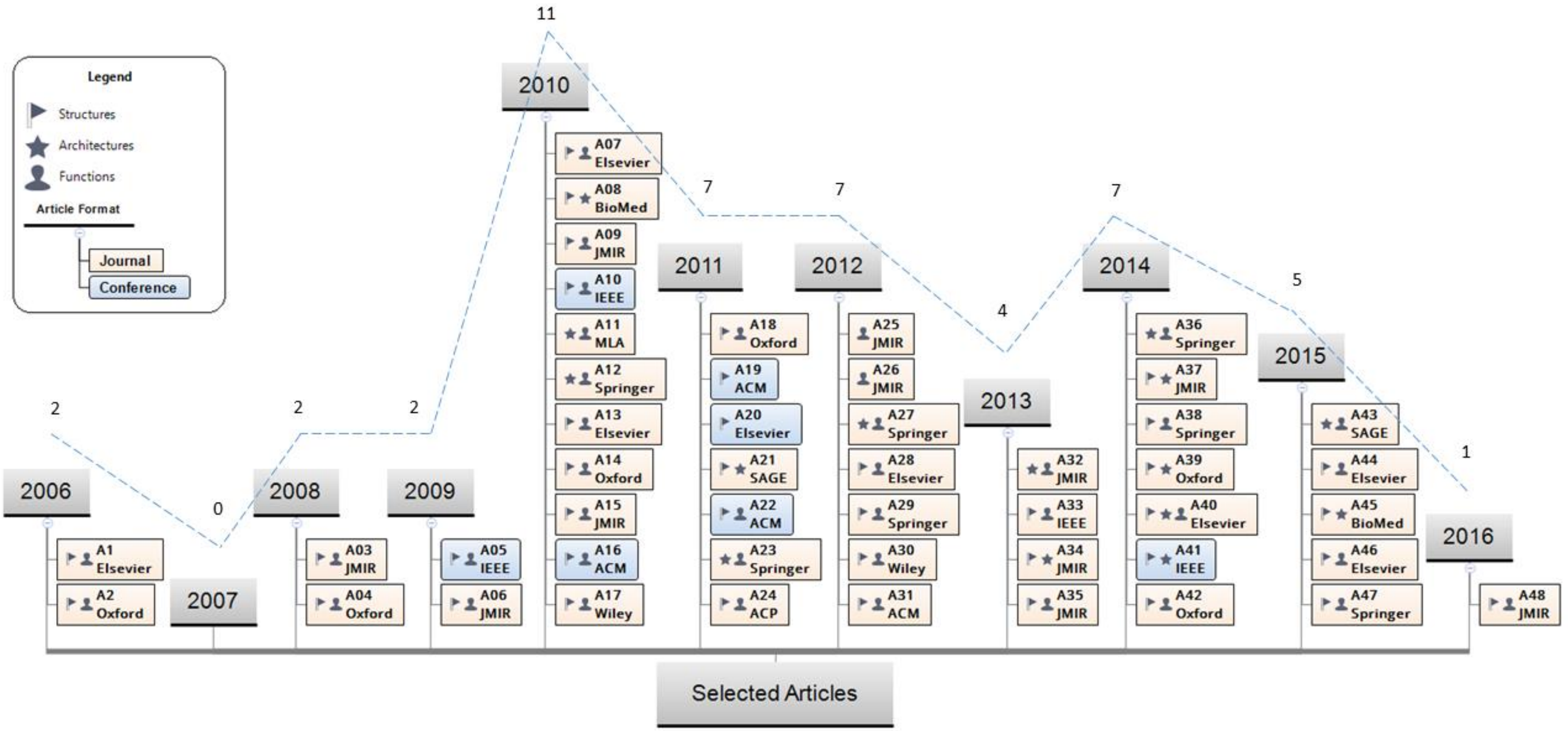

PHR Taxonomy

Group / Item	Description
**1. Structures**	Main data types and standards employed in health records.
1.1. Data Types	Data types found in PHRs (see subdivision and references in **SQ1**).
1.2. Standards	Standards to which PHRs can adhere (see subdivision and references in **SQ2**).
**2. Functions**	Depicts the main goals and features present in the PHRs.
2.1. Users/Profiles	User types and profiles that interact (see subdivision and references in **SQ3**).
2.2. Interaction	Patient's interaction types with a PHR (see subdivision and references in **SQ4**).
2.3. Data Source	Information techniques for input (see subdivision and references in **SQ5**).
2.4. Goals	Represents the aim of the PHR (see subdivision and references in **SQ6**).
**3. Architectures**	Architecture types and scopes (see subdivision and references in **SQ7**).
3.1. Models	Describes the main architectures models.
3.2. Coverage	Has a physical location division for data.

Challenges and Open Questions

Group/ID	Challenge and Concern (CC)
**GCC1 – Collaboration and Communication**	
CC01	Context-aware computing
CC02	Wearable computing, IoT
CC03	AI applied to health
CC04	Personalization, usability, familiarity, comfort
CC05	Manage medications
CC06	Patient-generated data
**GCC2 - Privacy, Security and Trust**	
CC07	Confidentiality and integrity
CC08	Data repository ownership
CC09	Authorization and access control technologies
CC10	Secure transport protocol
**GCC3 - Infrastructure**	
CC11	Portability – devices, equipment, HW
CC12	Efficiency and scalability
**GCC4 - Integration**	
CC13	Patterns in collecting medical data
CC14	Terminology
CC15	Interoperability

PHR Data Types

Type	Description
Allergies	Allergies and adverse reactions
Demographic	Patient statistics and clinical data
Documents	Attached files (photos, scanned docs)
Evolution	Progress and clinic notes, care plan
Family history	Family medical history
General	Patient registration information, emergency contact
Genetic	Genetic information
Home monitor	Home-monitored data
Immunizations	Immunization records (Vaccine), tracking immunizations
Insurance	Insurance plan information, coding for billing
Lab results	Laboratory and imaging test results (laboratory tests)
Major illnesses	List of major diseases
Medications	Medication list prescribed, past medicines taken
Prescriptions	Medical prescription refills (renewing)
Prevention	Preventive health recommendations
Providers	Previous healthcare provider list
Scheduling	Appointments, past procedures, Hospitalizations
Social history	Social history, lifestyle (health habits)
Summaries	Admissions, permanencies and discharges
Vital Signs	Status of bodily functions

PHR Related Standards

Group / Std.	Description
**GS1 – Nomenclature and Terminology**	
HNA / NIC	Nursing activities and intervention classification.
ICDx	International classification of diseases.
LOINC	Logical observation identifier names and codes.
SNOMED-CT	Terminology collection of medical terms.
UMLS	Unified medical language system.
**GS2 – Privacy**	
HIPAA	Health insurance portability and accountability act.
**GS3 - Structural and Semantic**	
ASC X12N	Accredited Standards Committee X12-INS.
CCD	Continuity of care document.
CCR	Continuity of care record.
CDA	Clinical document architecture.
DICOM	Digital imaging and communications in medicine.
EN 13606	EHR standards in Europe.
HL7 / FHIR / SMART	High Level 7 / FHIR (Fast Healthcare Interoperability Resources) / SMART (Substitutable Medical Apps).
ISO	TR (Technical Report) 14292 (PHR)<br>ISO/IEEE 11073 Personal Health Data (PHD).
OpenEHR	Open standards specification in e-health.
xDT	German family of data exchange formats
**GS4 – Templates and Technology Platforms**	
OpenMRS	Open Medical Record System
OSCAR	Open Source Clinical Application Resource.

PHR Profiles, Interaction Types and Goals

Profiles

- *Physician/Doctor*
- *Nurse*
- *Administrative*
- *Patient / Consumer*
- *Relative*
- *Public / Anonymous*

Interaction Types

- *Direct*
- *Indirect*
- *Outsourced*

Goals

- *Consult*
- *Maintain*
- *Monitor*

PHR Information Techniques

Technique / Profiles (Actors)	Description
**Data Collaboration (T1)**	
Health Professionals	Collaboration between multiple healthcare professionals. Healthcare providers are the owners (paternalistic relationship).
**Patient Reports (T2)**	
Patient	Patient reports data, e.g., listing drugs that are being used or menstrual period data.
**Adaptive Platforms (T3)**	
Environment	Aggregate sources provisioning individualized personal eHealth services combined with context info, including monitoring sensors. Patient and providers share the fill.
**Anonymization (T4)**	
Anonymous	Anonymizing social network data.

PHR Architectures

Group / Item	Description
**AG1 – Models**	
On paper	health records are kept on paper
Inside	PHR is kept in local repositories, inside provider, for example
Outside	PHR is distributed or shared between servers outside the provider
Hybrid	PHR is distributed inside and outside of the provider
**AG2 – Coverage**	
Standalone	data coverage is used only in the provider area
Local	area is at the city level
Regional	data are used in the state or province
National	coverage encompasses the nation
International	coverage transcends the nation

Discussion

- Overview of the technology regarding PHR in the last ten years from a number of candidate articles.
- Identified several common aspects of studies by answering a number of research questions.
- Proposed a PHR taxonomy and identified gaps to be further researched that represent challenges and issues
- Identified data types, standardization, profiles and a classification of input techniques

Limitations

- Review focused exclusively on articles addressing the inherent PHR concepts.
- Research sought to answer the resolution of the research questions
- Research was limited to obtaining articles published in a number of scientific portals related to ICT and health.
- Research was reduced to studies found from these websites when we implemented the steps of the SLR Methodology.
- Focused on scientific articles and did not address commercial or more technological approach solutions.

Conclusions

- This study aimed to raise and discuss the main issues regarding PHR and identify the concepts of the technology in this area.
- To answer the research questions throughout this work, we sought first to systematize and qualify the information that served as a source for the survey.
- For the completion of the work, we were able to identify and propose a broad taxonomy for the scope of work, which was created after an analysis of the relevant articles in last decade.
- All of these results contribute to the achievement of a significant coverage degree regarding the technology related to PHRs.
